# Assessment criteria and risk mitigation of hydrogeothermal energy portfolios for district heating

**DOI:** 10.1038/s44172-025-00478-3

**Published:** 2025-07-30

**Authors:** Michael C. Drews, Daniela Pfrang, Felix Schölderle, Kai Zosseder

**Affiliations:** 1 Technical University of Munich, Munich, Germany; 2 SWM Services GmbH, Munich, Germany

**Keywords:** Geothermal energy, Energy economics

## Abstract

In heating-intensive areas, low-to-intermediate temperature hydrogeothermal energy (depth >1500 m below ground level, temperature <200 °C) has the potential to replace fossil fuels in the heating sector. One of the biggest obstacles to the wide-spread implementation of hydrogeothermal energy is the exploration risk, the probability of finding geological conditions, which do not yield long-term economic thermal power. Here, we develop and investigate different assessment criteria of potential hydrogeothermal projects to minimize this exploration risk and the associated economic consequences in an inventory-portfolio approach. To do so, we combine a simplified inventory-portfolio approach with uncertain and spatially varying subsurface parameters and a cost model in a Monte Carlo simulation framework. We use an established hydrogeothermal energy play in SE Germany as an example and evaluate the performance of the tested assessment criteria vs. average exploration risk, total produced energy, total cost and cost of failure due to non-discoveries. Our results demonstrate that careful selection of formalized assessment criteria is key to mitigate exploration risk. We conclude that a holistic top-down planning approach, which combines the comprehensive and standardized characterization of geological and economic conditions on geothermal play-scale, is necessary to effectively employ hydrogeothermal energy as a replacement of fossil fuelled heating.

## Abstract (web summary)

Michael Drews et al. investigate the impact of drilling order criteria on the economics of hydrogeothermal heating portfolios. They show that considering cost, thermal power and geologic uncertainty can minimize the risk profile and cost of failure.

## Introduction

Deep geothermal energy harvests the heat stored in the Earth’s subsurface to directly use it for heating or to convert it into electricity. To do so, different concepts exist, which either mine the heat by producing existing hot thermal water from porous, karstified or fractured rock formations (hydrogeothermal energy) or from the rock itself^1,2^. The latter requires to either produce or enhance permeability artificially (e.g., via hydraulic stimulation), typically known as enhanced geothermal systems^3^, or to drill large heat exchanger systems, commonly known as advanced geothermal systems^4,5^. In contrast, hydrogeothermal energy requires the presence of an aquifer with natural permeability and thermal water. For all these systems, CO_2_ can also be considered as a working fluid to further improve the thermal power output^4,6,7^. Here, we focus on deep (>1500 m below ground level) low-to-intermediate temperature (<200 °C) hydrogeothermal energy, which is an established, but still not widely used technology^8^ with the potential to replace fossil-fueled energy—not only for electricity generation, but also for district heating^9^.

Several countries have identified hydrogeothermal district heating as a key factor to master the transition from fossil-fueled heating to renewable heating^10–12^. Development of hydrogeothermal energy requires i) non-invasive geophysical exploration of the subsurface to locate a potential thermal aquifer, ii) drilling, logging and testing of a first well and, if testing proved successful, iii) drilling of a second well and subsequent building of the heat plant and distribution infrastructure. One well is then utilized to produce the thermal water from the aquifer, while the second well serves the purpose of reinjecting the cooled thermal water into the aquifer after passing through the heat exchanger, which transfers the heat to the working fluid of the district heating grid. This simple setup is commonly known as a hydrogeothermal doublet^1,2^. The long-term (e.g., over an amortization period of 30 years) thermal power of a hydrogeothermal doublet depends on the volumetric heat capacity, a sustainable volumetric production rate and the difference between the production temperature and reinjection or flowback temperature of the thermal water^1,2^.

With a few exceptions (e.g., Iceland) and despite its rather simple setup, the current share of hydrogeothermal energy in the heating mix of heating-intensive areas of the world is usually well below 1%^9^. The main obstacle to a wider spread implementation is the disadvantageous cost-risk profile of hydrogeothermal energy^9,13^. 30–70% of the total capital expenditure must be spent before the economics and productivity of the hydrogeothermal project are known, which is typically the case after drilling and testing of the first well^1,13,14^. The risk of discovering a thermal aquifer, which yields economically insufficient thermal power, is called exploration risk. The exploration risk is defined by a preset economic expectation of thermal power in relation to the pre-drill uncertainty around expected production temperatures and volumetric production rates, both of which depend on geological properties of the thermal aquifer. Here, volumetric production rates depend on many parameters (e.g., hydraulic properties and thickness of the thermal aquifer), and even though hydraulic or chemical stimulation might improve productivity, volumetric production rates usually display the highest sensitivity to performance^15,16^. In contrast, production temperature often can be reasonably predicted by temperature-depth gradients of previously drilled wells, but still exhibits uncertainties. These geological uncertainties must be assessed and described by statistical distributions and probabilities^17–19^.

Geophysical surveys and geological modeling are employed to reduce geological uncertainties and to better understand the exploration risk prior to drilling, but, due to aquifer heterogeneity, irreducible uncertainty and risk remain until the first well has been drilled and tested and to a lesser degree even beyond (e.g., regarding the risk of thermal breakthrough or induced seismicity). The exploration risk, therefore, must be covered through an inventory-portfolio approach, where successful projects compensate the exploration cost of unsuccessful projects^20^. Hereby, the drilling order of the prospective projects (prospects) is key to minimize the overall exploration risk and cost of failed projects as well as to maximize the total thermal power of the portfolio. As previously established in conventional oil and gas exploration, the best prospects should be drilled first and assessment criteria to rank potential prospects of a drilling portfolio are critical^20,21^. However, most geothermal plays are still developed on a stand-alone, “first-come, first-serve” basis^20^.

In this contribution, we develop and test different assessment criteria for hydrogeothermal inventories for district heating. To reduce complexity, we assume a simple hydrogeothermal doublet design, constant thermal power throughout the amortization period and geological uncertainty only around volumetric production rates prior to drilling of the first well. We apply a cost model for direct and in situ heat use of hydrogeothermal doublets. We employ this framework in a Monte Carlo simulation approach, which we apply to the well-studied and already established hydrogeothermal energy play of the North Alpine Foreland Basin in SE Germany as a real-world example. To be able to consider a sufficient number of prospects, we conduct this simulation for comprehensive deep geothermal use without spatial limitations such as demand, infrastructure or environmental protection zones. We also assume steady-state conditions, regarding both physical (reservoir heat depletion, production flow rate reductions, chemical reactions that can lead to clogging, etc.) and non-physical (i.e., economic, such as inflation rate) parameters. We evaluate the performance of the tested assessment criteria vs. average exploration risk, total produced energy, total cost and cost of failure due to non-discoveries. We demonstrate that a risk-adjusted assessment criterion performs best and formulate general recommendations for the future development of hydrogeothermal energy inventories.

## Results

### Assessment criteria for hydrogeothermal energy

Exploration risk, geological uncertainty and high upfront investments make hydrogeothermal energy exploration and development comparable to oil and gas exploration and development, where usually an inventory-portfolio approach is utilized^21,22^. However, most hydrogeothermal projects are still developed on a stand-alone and “first come, first serve” basis^20^ and an inventory-portfolio approach for hydrogeothermal play development has only been suggested recently^20,23,24^.

**Fig. 1:**
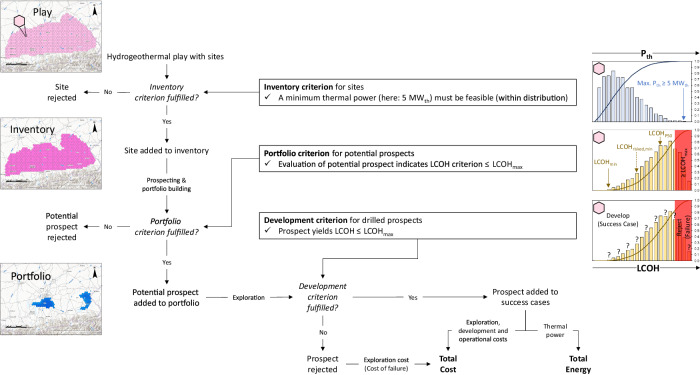
Simplified hydrogeothermal inventory-portfolio approach. The shown approach and decision chart also reflects the modeling framework employed in this study. *LCOH*  = levelized cost of heat, *LCOH*_*max*_  =  maximum tolerable *LCOH*.

A simplified inventory-portfolio approach adapted to hydrogeothermal energy is shown in Fig. 1. The local resource base is thereby given by a hydrogeothermal energy play with potential project locations (sites). A play is defined by a geographically constrained geological setting with a geothermal potential^25^. Its sites can be added to an inventory, if they fulfil the inventory criterion—for e.g., having the potential to yield or exceed a pre-defined thermal power (Fig. 1). The inventory is feeding the portfolio of sites to be drilled (prospects). To become a prospect, a potential prospect from the inventory must fulfil the portfolio criterion (Fig. 1). While in the hydrocarbon industry a prospect must have the potential to yield a certain volume of hydrocarbons with an acceptable probability, a successful hydrogeothermal energy project should yield a thermal power which can be maintained over a predefined amortization period with an acceptable capital and operational expenditure. The levelized cost of heat *LCOH*, which describes the cost per MWh produced, reflects these standards and is frequently used^15,26^.

Depending on the geological uncertainties, each potential prospect has a distribution of possible *LCOH* outcomes driven by the geological uncertainties^15,17–20^. Geophysical and geological investigations serve the sole purpose of reducing these uncertainties prior to drilling of the first well^20^. Typically, a maximum tolerable levelized cost of heat *LCOH*_*max*_ is defined. Any higher *LCOH* would therefore be an economic failure. *LCOH*_*max*_ is also the benchmark for adding a potential prospect to the drilling portfolio (portfolio criterion) and if the drilled prospect is a success case which can be developed (development criterion). The cost for exploration, development and operation of a successful case is added to the total cost of developing the hydrogeothermal play, and the produced energy is added to the total energy output. If the prospect does not fulfil the development criterion, the prospect is rejected and only the cost of failure (exploration cost) is added to the total cost of the portfolio (Fig. 1).

A key question is, which *LCOH* is selected from the distribution of possible *LCOH* outcomes of each potential prospect for comparison with *LCOH*_*max*_ (portfolio criterion, Fig. 1). An optimistic selection of *LCOH* to test the portfolio criterion would be the minimum expectable (lowest possible) levelized cost of heat *LCOH*_*min*_, regarding all considered uncertainties. Likewise, the median (50th percentile or *P50) LCOH*_*P50*_ or any other percentile of the distribution of the potential prospect’s levelized cost of heat could be used. However, these *LCOH* values do not consider the probability of failure (exploration risk) and the cost of failure. Ideally, the potential cost of failure is included in relation to the producible energy weighted by the exploration risk and probability of success, respectively. The risk-adjusted levelized cost of heat *LCOH*_*risked*_ fulfills these conditions and can be calculated by assuming an expected monetary value with zero-profit (break-even) *LCOH*, which is essentially a risk-weighted mean value of *LCOH*^20,21,27^.

**Fig. 2:**
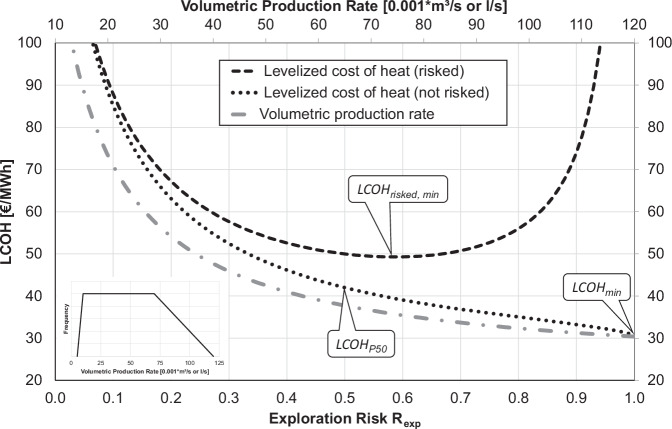
Comparison of portfolio criteria. Comparison of different portfolio criteria applied to a potential prospect with fixed production temperature and cost but uncertain volumetric production rate *Q* (the inset on the lower left shows the exemplary frequency distribution of *Q*). Displayed are the levelized cost of heat *LCOH* (vertical axis) as a function of the exploration risk *R*_*exp*_ (horizontal axis). *LCOH* are calculated using a previously published cost model^26^ for different volumetric production rates from the frequency distribution (not risked *LCOH*  =  black dotted line) and when including the cost of failure, if the prospect was rejected (*LCOH*_*risked*_  =  black dashed line). Highlighted are the minimum and median expectable not risked levelized cost of heat *LCOH*_*min*_ and *LCOH*_*P50*_, respectively, and the minimum of the risk-adjusted levelized cost of heat *LCOH*_*risked, min*_.

In contrast to *LCOH* criteria which reflect an arbitrary percentile of the *LCOH* distribution (e.g., *LCOH*_*min*_ or *LCOH*_*P50*_), *LCOH*_*risked*_ incorporates the cost of failure as a function of the exploration risk. Consequently, *LCOH*_*risked*_ vs. exploration risk follows an asymmetric parabola (Fig. 2): for low exploration risks, the cost of failure weigh little, and the thermal power is achieved with high probability, but can be low. Accordingly, high values of *LCOH*_*risked*_ would be accepted. If accepting high exploration risks, the expected thermal power would be high but achieved with low probability. In addition, the exploration costs in case of prospect rejection weigh more and *LCOH*_*risked*_ will again yield high values. Consequently, there is an exploration risk, where *LCOH*_*risked*_ becomes minimal (Fig. 2). This minimum risk-adjusted levelized cost of heat *LCOH*_*risked, min*_ is therefore an optimized measure of the quality of the prospect.

### Application of assessment criteria to a real-world example

As a portfolio criterion, we test the *LCOH*_*risked, min*_ criterion in the hydrogeothermal energy play of the North Alpine Foreland Basin in SE Germany and compare its performance with *LCOH*_*min*_ and *LCOH*_*P50*_. More than 30 hydrogeothermal sites have been drilled with an overall exploration risk of ~20% in the North Alpine Foreland Basin^28,29^. Projects are typically designed as doublets and produce from a Mesozoic karstified carbonate aquifer^30,31^. The produced thermal water is primarily used for district heating via heat exchangers and preexisting, originally fossil-fueled heating grids. In some cases, electricity is also generated through a binary cycle. The flowback or reinjection temperature is typically around 60 °C, independent of the production temperature^29^. Although the play is already the most developed hydrogeothermal energy play in Germany, expansion of the total thermal power is planned in the next decades^29^. For example, the city of Munich located in the center of the study area committed to cover its entire heat demand largely through hydrogeothermal energy until 2040^32^.

**Fig. 3:**
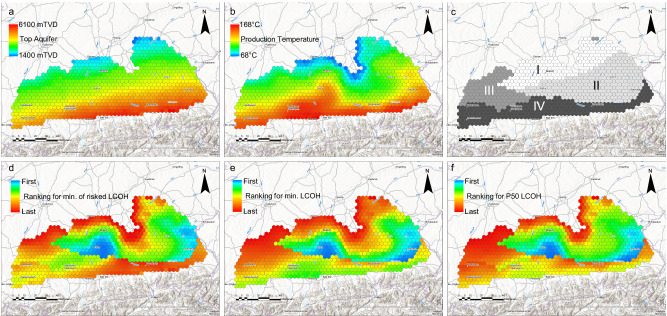
Spatial distribution of aquifer properties and portfolio criteria. **a**True vertical depth below ground level in meters (TVDm) of the top of the thermal aquifer^33,44^. **b** Production temperature in °C calculated from previously published temperature data^33,34,44^ and using a correction based on production data. **c** Zones of equal volumetric production rates modeled with trapezoidal distributions described by minimum, beginning of plateau, end of plateau and maximum volumetric production rates in l s^−1^ (zone I: 20-110-150-180, zone II: 5-80-150-180, zone III: 0-10-50-70 and zone IV: 0-0.1-30-70). **d** Spatial distribution of the minimum of risk-adjusted levelized cost of heat *LCOH*_*risked, min*_. **e** Spatial distribution of the minimum levelized cost of heat *LCOH*_*min*_. **f** Spatial distribution of the median levelized cost of heat *LCOH*_*P50*_. Color-coding of portfolio criteria indicates their position in the ranking, with blue indicating the first (best and therefore lowest *LCOH*) ranks and red indicating the last (worst and therefore highest *LCOH*) ranks. Background terrain and reference map by Esri, Geoland, NASA, NGA, USGS, with sources from Esri, TomTom, Garmin, GAO, NOAA, USGS, © OpenStreetMap contributors, and the GIS User Community.

Aquifer temperature, and thus production temperature, generally follows the N-S aquifer depth trend^33^ (Fig. 3a), and reaches temperatures of up to 170 °C along its southern border^33,34^. Isotherms follow a WSW-ENE trend; however, elevated aquifer temperatures are present to the immediate south of Munich and are lower eastward of Munich^33,34^ (Fig. 3b). Volumetric production rates can vary from less than 10 l/s up to 200 l/s and depend on the hydraulic properties of the aquifer^18,31,35–43^. Hereby, volumetric production rates are lowest in the deepest and hottest part of the aquifer, but due to sedimentological variations are also less favorable in the western part of the study area. Accordingly, the hydrogeothermal aquifer can be divided into four zones of equal volumetric production rate distributions, which reflect the uncertainty within these zones (Fig. 3c).

For our real-world example, we divided the area covered by the carbonate play into 845 hexagons. Each hexagon must have the potential to yield a maximum thermal power of ≥5 MW_th_ (inventory criterion, cf. Fig. 1) by means of a single doublet and covers an area of 10 km^2^ with a diagonal length of 4 km. This area reflects the common practice in the study area that hydrogeothermal projects should be at least 3 km apart from each other to avoid hydrothermal breakthrough between projects during production. We treat each hexagon as a potential prospect of the inventory and calculate *LCOH*_*risked, min*_, *LCOH*_*min*_, and *LCOH*_*P50*_ as portfolio criteria for each hexagon (Fig. 3d–f). For the calculation of thermal power and *LCOH* we use the averages of previously published aquifer depths^33,44^, a correction of the available aquifer temperature^33,34,44^ to production temperature, using operational data from 33 wells, a flowback temperature of 60 °C, based on the same operational data and published data from ref. ^29^, volumetric production rate distributions (Fig. 3a–c) and a previously published cost model, which was specifically developed for hydrogeothermal energy development in SE Germany^26^. The cost model accounts for exploration costs (geophysical surveying, project management, drilling and testing of the first well), development costs (drilling of the second well, cost of the pump, heat exchanger, seismic monitoring, project management) and operational costs (electricity, maintenance, insurance and personnel). Hereby, the cost model also considers dependencies between aquifer properties and cost items, e.g., drilling cost is calculated as a function of aquifer depth, the capital and operational expenditure of the pump and cost of the heat exchanger depend on volumetric flow rates and production temperatures, respectively. Input parameters such as operational hours per year, electricity price, interest rate and pump depth are kept constant.

**Fig. 4:**
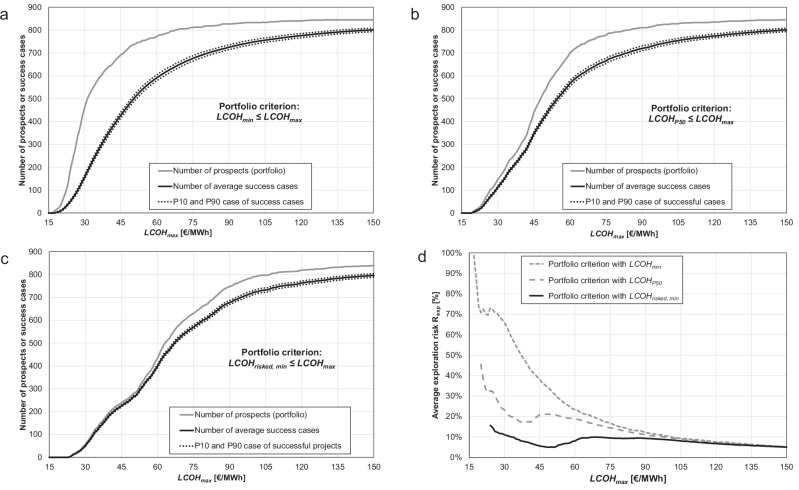
Comparison of portfolio criteria as a function of the maximum tolerable levelized cost of heat. **a** Portfolio size and number of success cases (average, 10th percentile P10 and 90th percentile P90) as a function of the maximum tolerable levelized cost of heat *LCOH*_*max*_, if the minimum expectable levelized cost of heat *LCOH*_*min*_ is used as portfolio criterion. **b**Portfolio size and number of success cases (average, 10th percentile P10 and 90th percentile P90) as a function of *LCOH*_*max*_, if the median (P50) expectable levelized cost of heat *LCOH*_*P50*_ is used as portfolio criterion. **c** Portfolio size and number of success cases (average, 10th percentile P10 and 90th percentile P90) as a function of *LCOH*_*max*_, if the minimum of the risk-adjusted levelized cost of heat *LCOH*_*risked, min*_ is used as portfolio criterion. **d** Comparison of the average exploration risk as a function of *LCOH*_*max*_ between the portfolio criteria *LCOH*_*min*_, *LCOH*_*P50*_ and *LCOH*_*risked, min*_.

**Fig. 5:**
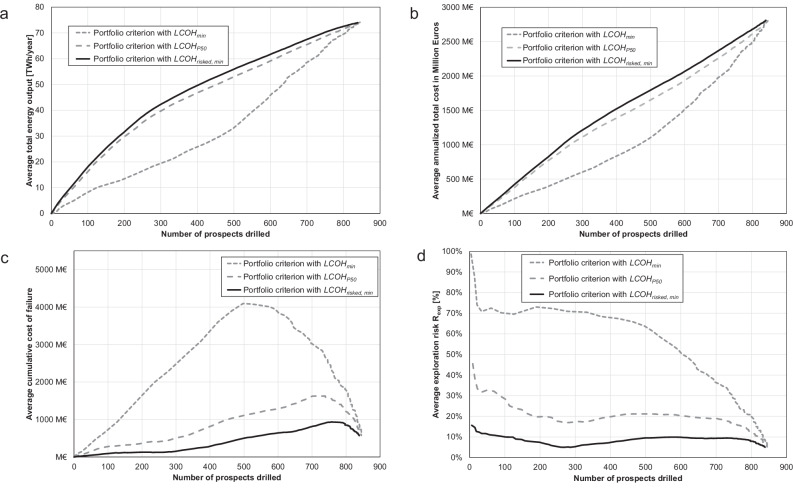
Comparison of portfolio criteria as a function of portfolio size or number of prospects drilled. **a** Comparison of the average total energy output of the portfolio as a function of number of prospects drilled between *LCOH*_*min*_, *LCOH*_*P50*_ and *LCOH*_*risked, min*_. **b** Comparison of the average annualized total cost of the portfolio as a function of number of prospects drilled between *LCOH*_*min*_, *LCOH*_*P50*_ and *LCOH*_*risked, min*_. **c** Comparison of the average cost of failure of the portfolio as a function of number of prospects drilled between *LCOH*_*min*_, *LCOH*_*P50*_ and *LCOH*_*risked, min*_. **d** Comparison of the average exploration risk of the portfolio as a function of number of prospects drilled between *LCOH*_*min*_, *LCOH*_*P50*_ and *LCOH*_*risked, min*_. *LCOH*_*min*_=  minimum expectable levelized cost of heat, *LCOH*_*P50*_ = median (P50) expectable levelized cost of heat and *LCOH*_*risked, min*_ = minimum of the risk-adjusted levelized cost of heat.

We then run a Monte Carlo simulation, which combines the decision flowchart in Fig. 1 with the cost model^26^. To create an order in which the best prospects are tested first, we successively increase *LCOH*_*max*_ and reject all potential prospects with a *LCOH* criterion that exceeds *LCOH*_*max*_ (portfolio criterion, cf. Fig. 1). We then use *LCOH*_*max*_ for the development criterion and only develop a prospect if its simulated *LCOH* is less than *LCOH*_*max*_. We investigate the Monte Carlo simulation results for portfolio size, number of success cases and exploration risk as a function of *LCOH*_*max*_ (Fig. 4a–d). Since the portfolio size or number of prospects monotonically increases with increasing *LCOH*_*max*_, we are also able to investigate the average total energy, average total cost, cumulative cost of failure and the exploration risk for each portfolio criterion as a function of the number of prospects (Fig. 5a–d).

*LCOH*_*min*_ is the most optimistic measure of the quality of a prospect, but the results show that it also performs poorly: *LCOH*_*min*_ has the largest portfolio size (number of prospects or wells drilled) and number of success cases for each defined *LCOH*_*max*_ (Fig. 4a), but also the highest exploration risk and therefore number of failure cases, the lowest total energy output, lowest total cost, and the highest cumulative cost of failure (Fig. 4d and Fig. 5a–d). The lowest total cost is the result of a high number of failure cases, whose costs for development and operations are not spent. Similarly, to *LCOH*_*min*_, *LCOH*_*P50*_ is an arbitrary cutoff from the pre-drill estimate of the *LCOH* distribution of each potential prospect. However, *LCOH*_*P50*_ is a more conservative measure of the quality of a potential prospect and performs better than *LCOH*_*min*_. Since *LCOH*_*P50*_ is naturally closer to the balance point between thermal power, cost and exploration risk (cf. Fig. 2), the number of failure cases is reduced (Fig. 4b, d) and with it the total energy output and total cost are increased. Compared to *LCOH*_*min*_, the cumulative cost of failure normalized to the number of drilled prospects is reduced by up to a factor of 4 (Fig. 5a–d).

The exploration risk associated with *LCOH*_*risked, min*_ varies for each potential prospect but minimizes the overall exploration risk of the portfolio. In particular, for low and realistic *LCOH*_*max*_ values, *LCOH*_*risked, min*_ performs better than *LCOH*_*min*_ and *LCOH*_*P50*_. The portfolio size for each defined *LCOH*_*max*_ is reduced, but the number of failure cases, the overall exploration risk, total energy output and cumulative cost of failure are optimized, because *LCOH*_*risked, min*_ reflects the best cost-energy-risk ratio for each potential prospect (Fig. 4c, d and Fig. 5a–d). Depending on the defined *LCOH*_*max*_, the cumulative cost of failure normalized to the number of drilled prospects for *LCOH*_*risked, min*_ can be up to an order of magnitude lower than for *LCOH*_*min*_.

Using *LCOH*_*risked, min*_ as portfolio criterion, more than 50% of the theoretical total thermal output (74 TWh year-1) can be achieved with drilling less than 30% of all prospects (246) and with a cumulative cost of failure of less than 125 M€, compared to 64% (539) and more than 4000 M€ or 32% (270) and more than 420 M€ for *LCOH*_*min*_ and *LCOH*_*P50*_, respectively (Fig. 5a–d). Only beyond a *LCOH*_*max*_-cutoff of 100 € MWh-1, the overall exploration risk profiles of the three tested portfolio criteria start to converge (Fig. 4d).

## Discussion

Hydrogeothermal projects are often developed on a stand-alone basis following a “first-come, first-serve” approach. However, our study demonstrates that an inventory-portfolio approach should be followed and that the selection of formalized assessment criteria for hydrogeothermal prospects is critical. An assessment criterion that includes the thermal power, cost and potential cost of failure as a function of the exploration risk is recommended over standard percentile cutoffs of the pre-drill distribution of possible levelized cost of heat of each potential prospect. Thereby, it is key to constrain and minimize the geological uncertainties in a standardized and comparable way across the hydrogeothermal play. However, the median value is a reasonable and easy-to-estimate alternative, because the best thermal power vs cost vs risk ratio is typically given for intermediate percentile cutoffs. It is in particular important to select the right portfolio criterion at the beginning of the development of a hydrogeothermal play; here, the demand for success cases and risk aversion are typically highest and it is critical to develop the “low hanging fruits” first.

In our real-world example from a hydrogeothermal energy play in SE Germany, the reinjection temperatures are around 60 °C for residential district heating^29^, this temperature is consistent across both solely heating plants and electricity-producing plants. Consequently, the ideal depth to achieve minimum *LCOH* is largely controlled by the geothermal gradient within the zones of equal flow rate distribution. The prospects with the potentially lowest *LCOH* are therefore directly southward of Munich where the thermal aquifer depth is between 3000 and 4000 m. However, we would like to point out that in geothermal plays, where the latest generation of district heating networks is available and connected to energy-optimized building systems that minimize the return temperature, drilling to shallower depths might eventually be more efficient and economical.

Application of an assessment criterion which combines thermal power, cost and the exploration risk to the real-world example showed that 50% of the inventory’s total thermal energy could be tapped with an average levelized cost of heat of less than 30 € MWh-1. Hereby, less than 30% of the inventory would have to be drilled with a cumulative cost of failure which is reduced by a factor of 30 compared to only assessing potential prospects on their theoretical best performance, which would be similar to a stand-alone “first-come, first-serve” development approach.

Even though we only consider a simplified hydrogeothermal play development with doublets, our study demonstrates that hydrogeothermal energy can be a very competitive green alternative for district heating, and its exploration risk can be minimized to an acceptable degree if a top-down inventory-portfolio planning approach is followed. The shown approach is likely also extendable to electricity production and applicable to other geothermal technologies such as enhanced and advanced geothermal systems, both of which also depend on the uncertain geologic conditions of the subsurface. Implementation of such a top-down inventory-portfolio approach could be implemented either by government or non-government exploration risk insurance solutions or by larger companies or consortia of companies, which can afford the development of large hydrogeothermal inventories. In order to minimize geological uncertainties, comprehensive, upfront geophysical and geological exploration and investigations are necessary for all potential prospects of a portfolio. If the preliminary investigations are bundled, approval procedures can also be combined, mobilization and demobilization costs can be saved, and better prices and faster implementation can be achieved. In addition, if the assessment of the inventory is updated after drilling new wells, geological uncertainties can be additionally reduced and the exploration risk further mitigated. Further potential can be unlocked if coupling of heating grids for redundancy and peak load optimization, new geothermal technologies such as enhanced and advanced geothermal systems, additional thermal aquifers and coupling with other technologies (solar, shallow geothermal, seasonal aquifer thermal energy storage) are considered. Switching from a stand-alone development approach to an inventory-portfolio approach, therefore, opens the potential of scalability of hydrogeothermal energy for district heating.

## Methods

### Calculation of thermal power *P*_*th*_

The thermal power *P*_*th*_ depends on the volumetric heat capacity of the thermal water *c*_*w*_, the volumetric flow rate *Q* and the extracted heat, which is described by the difference between the temperature of the produced thermal water *T*_*prod*_ and the flowback temperature of the thermal water after heating usage *T*_*back*_^1^:

1 $${P}_{{th}}={c}_{w}\cdot Q\cdot \left({T}_{{prod}}-{T}_{{back}}\right)$$

where *P*_*th*_ is usually given in MW_th_, *c*_*w*_ can be assumed constant at 4.2 MJ m^−3^ K^−1^, *Q* is provided in m^3^ s^−1^ and *T*_*prod*_ *– T*_*back*_ in K.

### Calculation of levelized cost of heat *LCOH*

Levelized cost of heat *LCOH* for a hydrogeothermal energy project is calculated in cost per energy unit, e.g., € MWh-1^15^:

2 $${LCOH}=\frac{{C}_{{Project}}}{{E}_{{Project}}}$$

Where *E*_*project*_ is the annual energy produced by a hydrogeothermal energy project:

3 $${E}_{{Project}}={P}_{{th}}\cdot L$$

*L* is the annual number of operating hours (load). *C*_*project*_ is the sum of all operational expenditures (*OPEX*) per year and all annualized capital expenditures *CAPEX*, which are calculated by multiplying *CAPEX* with the annuity factor *a*:

4 $$a=\frac{{\left(1+i\right)}^{t}\cdot i}{{\left(1+i\right)}^{t}-1}$$

where *i* is the interest rate per year and *t* is the amortization period in years. In its simplest form, hydrogeothermal heat projects consist of a production well that produces thermal water from an appropriate geological formation (aquifer) using a thermal water pump, a heat plant (heat exchangers and heating grid) and an injection well to reinject the cooled thermal water back into the aquifer. For hydrogeothermal heat projects, *OPEX* is therefore mainly influenced by the energy required to drive the thermal water pump and to maintain the heat plant and production facilities (e.g., replacing the thermal water pump). On a first-order view, *CAPEX* of hydrogeothermal heat projects consists of exploration costs *CAPEX*_*exp*_ (geophysical surveys, geological studies, drilling, logging and testing of the first well) and development costs *CAPEX*_*dev*_ (drilling and testing of the second well, thermal water pump, heat exchangers and, if not already available, the heating grid). The total annualized cost *C*_*Project*_ therefore calculates as follows:

5 $${C}_{{Project}}={OPEX}+a\cdot {CAPEX}={OPEX}+a\cdot \left({{CAPEX}}_{\exp }+{{CAPEX}}_{{dev}}\right)$$

### Exploration risk and probability of success

Since the hydraulic and geological properties of the thermal aquifer, which drive the volumetric production rates of a hydrogeothermal heat project, and the temperatures in the subsurface cannot be exactly determined before drilling of the first well is completed, the thermal power and levelized cost of heat are uncertain and follow a distribution. In our study, we predefine a maximum tolerable levelized cost of heat *LCOH*_*max*_. The exploration risk *R*_*exp*_ is then the likelihood of finding aquifer properties, which yield a levelized cost of heat ≥*LCOH*_*max*_. The probability of success *POS* describes the likelihood to fall below *LCOH*_*max*_ and is directly related to *R*_*exp*_:

6 $${POS}=1-{R}_{exp }$$

### Risk-adjusted *LCOH*

While *CAPEX*_*exp*_ is always invested, *CAPEX*_*dev*_ and *OPEX* will only be spend, if the project is successful, meaning testing of the first well confirms a producible thermal water resource that yields *LCOH* ≤ *LCOH*_*max*_. In case of failure, *CAPEX*_*exp*_ becomes the cost of failure *COF*. Spending of *CAPEX*_*dev*_ and *OPEX* and producing hydrogeothermal heat are hence tied to a probability which equals *POS* or 1 – *R*_*exp*_. This relationship is formulated through the expected monetary value *EMV*^27^:

7 $${EMV}={POS}\cdot {NPV}+{R}_{exp }\cdot {COF}$$

Where *NPV* is the net present value. Assuming zero profit and incorporating *LCOH* into Eq. 7 implies that ${EMV}=0,{NPV}=\,{C}_{{Project}}-{LCOH}\cdot {E}_{{Project}}$ and ${COF}=a\cdot {{CAPEX}}_{exp }$, yielding:

8 $$0={POS}\cdot \left({C}_{{Project}}-{LCOH}\cdot {E}_{{Project}}\right)+{R}_{\exp }\cdot a\cdot {{CAPEX}}_{\exp }$$

Solving Eq. 8 for *LCOH* then allows for calculation of the risk-adjusted levelized cost of heat *LCOH*_*risked*_:

9 $${{LCOH}}_{{risked}}=\frac{{POS}\cdot {C}_{{Project}}+{R}_{\exp }\cdot a\cdot {{CAPEX}}_{\exp }}{{POS}\cdot {E}_{{Project}}}$$

Which, using Eqs. 5 and 6, can be rewritten to:

10 $${{LCOH}}_{{risked}}=\frac{{POS}\cdot \left(a\cdot {{CAPEX}}_{{dev}}+{OPEX}\right)+a\cdot {{CAPEX}}_{\exp }}{{POS}\cdot {E}_{{Project}}}$$

### Modeling framework for the hydrogeothermal energy play in the North Alpine Foreland Basin

The geological and hydraulic data for the analysis are the production temperatures at the wellhead, flowback temperatures, the depth of the hydrogeothermal aquifer and the volumetric production rates. We take the data from 33 wells, from the freely available geothermal information system GeotIS^33,34,44^ and data annually published by the Bundesverband Geothermie^29^. The spatial production temperature distribution is created as a raster with a cell size of 100 × 100 m and the production rates are postulated for four basic production zones I to IV^31,45^.

### Production temperature *T*_prod_

Available subsurface temperature models^33,34,44^ give the static temperature at specific depths, e.g., the top of the aquifer. In the absence of better data such temperatures are a good estimate for the production temperature, but in order to retrieve the actual production temperature heat loss through pumping the thermal water from the aquifer to the wellhead must be considered^46,47^.

To determine the average production temperature, a linear regression is established between the available production temperatures of 29 geothermal plants and 4 thermal bath wells and the static top aquifer temperatures^33,34,44^. We used this regression to correct the aquifer temperature to production temperature. We then use the calculated mean value of each hexagon to regionalize the production temperature.

### Volumetric production rate distributions

Long-year operational data are available at 24 wells in the study area. A volumetric production rate distribution is determined for each hexagon. For this purpose, we divide the study area into four zones representing different volumetric production rate distributions. A Monte Carlo simulation with 2000 trials is then performed to simulate the volumetric production rate of each hexagon. Hereby, we model the volumetric production rate distributions with a trapezoidal distribution:

11 $${if}\;{a}\le x < {b}\;{then}\,{p}=\frac{{\left(x-a\right)}^{2}}{\left(d+c-a-b\right)\cdot \left(b-a\right)}$$

12 $${if}\;{a}\le x < {b}\;{then}\;{p}=\frac{2\cdot x-a-b}{\left(d+c-a-b\right)}$$

13 $${if}\;{c}\le x\le {d}\;{then}\;{p}=\frac{1-{\left(d-x\right)}^{2}}{\left(d+c-a-b\right)\cdot \left(d-c\right)}$$

where *a*, *b*, *c*, and *d* are the minimum, start of the plateau (first mode), end of the plateau (second mode) and maximum of the trapezoidal distribution, respectively. *x* is a random value of the trapezoidal distribution and *p* is the likelihood to draw a value smaller than or equal to *x*. Table 1 shows the minimum, first mode, second mode and maximum of the volumetric production rate distributions for each zone.

**Table 1:** Minimum, first mode, second mode and maximum of the trapezoidal distributions for the volumetric production rate

Zone	Volumetric production rate [0.001*m^3^ s^−1^ or l s^−1^]			
	Minimum	First mode	Second mode	Maximum
I	20	110	150	180
II	5	80	150	180
III	0	10	50	70
IV	0	0.1	30	70

### Cost model

For calculation of costs for exploration *CAPEX*_*exp*_, development *CAPEX*_*dev*_ and operation *OPEX* of hydrogeothermal heat production in the SE German part of the North Alpine Foreland Basin, we use a previously published cost model^26^. Table 2 provides an overview of the individual cost calculations and dependencies, restructured for the three project phases, exploration, development and operation. Drilling costs are calculated using a previously published drilling cost function^26^, which is comparable to other drilling cost functions^35,48–50^ and requires the total depth of a well in true vertical depth below ground level *TVD*_*TD*_ as input. Based on 19 recent hydrogeothermal wells, which were drilled after 2007 in the study area, *TVD*_*TD*_ can be reasonably described by multiplying the true vertical depth of the top of the aquifer^33,44^ with a factor of 1.1106.

**Table 2:** Techno-economic framework^26^

Cost	Purpose	Amount/Equation
***K1***	***Exploration costs CAPEX***_***exp***_	***K1.1*** + ***K1.2*** + ***K1.3***
K1.1	Fixed costs	1,526,000 €
K1.2	Drilling of 1^st^ well and supervision as a function of the depth of the aquifer center in true vertical depth *TVD*_*TD*_	1.015 · 1.228 · exp(4.354 · 10^-4^ · TVD_TD_) · 10^6^
K1.3	Project management	8% (K1.1 + K1.2)
***K2***	***Development costs CAPEX***_***dev***_	***K2.1*** + ***…*** + ***K2.8***
K2.1	Fixed costs	356,000 €
K2.2	Drilling of 2nd well and supervision as a function of the depth of the aquifer center in true vertical depth *TVD*_*TD*_	1.015 · 1.228 · exp(4.354 · 10^−4^ · TVD_TD_) · 10^6^
K2.3	Feed pump with pumping power PP_i_ and pressure difference dP = 7 · 10^6 ^Pa corresponding to a water column dh = 700 m	PP_i_ · 11970 · PP_i_^-0.319^ + 45,000 €
	Pump power PP_i_	Q · dP · 1.15 · 10^−3^
K2.4	Casing and power cable for feed pump	dh · (0.022 · PP_i_ + 79)
K2.5	500 m piping system	500 · 60000 · Q
K2.6	Heating plant with heat exchanger with thermal capacity corresponding to the local geothermal potential *P*_th_ [kW] and specific investment costs *k*_he_ = 400€/kW, building and other components	1.05 · (*P*_th_ · *k*_he_)
K2.7	Project management	8% (K2.1+ … + K2.6)
K2.8	Seismic monitoring	155,000 €
***K3***	***Operational costs OPEX***	***K3.1*** + ***…*** + ***K3.7***
K3.1	Electricity consumption of feed pump with nominal power PP_i_ [kW], operation time in full-load L [h] and electricity price ep	PP_i_ · L · ep
K3.2	Electricity consumption of auxiliary machinery	10% K3.1
K3.3	Maintenance and repair of wells	0.5% (*K*1 + *K*2.1 + *K*2.2 + *K*2.8)
K3.4	Maintenance and repair of thermal water system	3% (K2.3 + K2.4 + K2.5)
K3.5	Maintenance and repair of heating plant	1% K2.6
K3.6	Insurance	0.6% (K2.3 + K2.4 + K2.5 + K2.6)
K3.7	Personnel as a function of the thermal output of the heat plant P_th_ [MW_th_]	225,000 · exp(5 · 10^−3^ · P_th_)

**Fig. 6:**
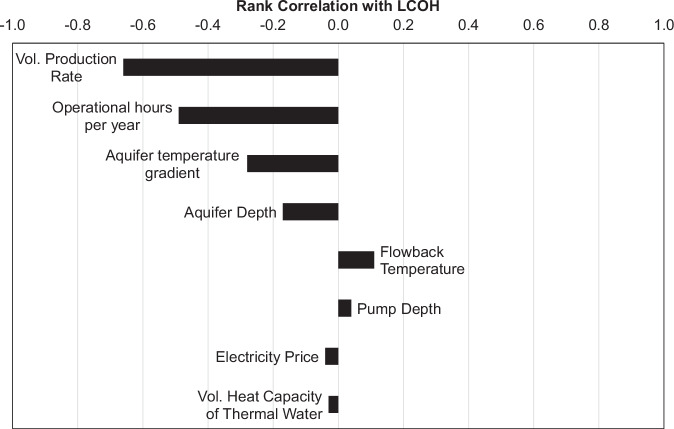
Sensitivity of Monte Carlo simulation approach. Sensitivities as rank coefficient of correlation of input parameters on levelized cost of heat *LCOH*. Parameter variations are 1–200 l s^−1^, 3000–8700 h year-1, 25–45 K km^−1^, 2000–5000  m, 40–60 °C, 100–900 m, 0.1–0.5 € kWh and 4.1–4.3 MJ m^−3^ K^−1^ for volumetric production rate, operational h per year, aquifer temperature gradient, aquifer depth, flowback temperature, pump depth, electricity price and volumetric heat capacity of the thermal water, respectively.

From Table 2 follows that in addition to top aquifer depth, production temperature and volumetric production rate, our analysis requires further input parameters as boundary conditions. We keep these input parameters constant and summarize them in Table 3. A sensitivity overview of all input parameters is shown in Fig. 6. Except for the annual operating hours *L*, which linearly scale with the produced energy *E*_*project*_, all constant input parameters have only a minor impact on the presented results. The largest impact is given by the volumetric production rate *Q*, which we vary both statistically and spatially, production temperature *T*_*prod*_ (here represented by the aquifer temperature gradient to maintain the temperature-depth relationship), which we vary spatially, and top aquifer depth, which we also vary spatially in our study. The flowback temperature *T*_*back*_ which we retrieved from operational data and annually published data^29^ also impacts the *LCOH*, but exhibits minor variation around the mean value of 60 °C in the real-world example from SE Germany.

**Table 3:** Constant input parameters and their values

Constant input parameter	Value
Hexagon size	10 km^2^
Flowback temperature *T*_*back*_	60 °C
Volumetric heat capacity of thermal water *ρc*_*w*_	4.2 MJ m^−3^ K^−1^
Pump depth *dh*	700 m
Operational hours of a heat plant per year *L*	7000 h
Electricity price *ep*	0.25 € kWh^−1^
Interest rate *i*	5%
Amortization period *t*	30 years

### Limitations

#### Thermal power and cost model

We assume constant thermal power over the amortization period. Even though several geothermal projects in the study area produce for more than two decades with constant thermal power, a thermal power decline over time should be expected, as the geothermal energy is almost always extracted at a higher rate than the natural rate of the Earth’s heat replenishment. For geothermal plays with high volumetric production rates, this effect becomes in particular important once the play development stage moves into a more mature state. The applied cost model is a simplification, and only considers simple geothermal doublets, while many projects in the study area are currently developed with a multi-doublet design and up to eight wells per project. We also keep many inputs constant (e.g., pump depth and electricity price) and neither consider inflation nor any cost uncertainties.

#### Spatial distribution of geothermal heat plants

In order to include a sufficient number of prospects, the Monte Carlo simulation assumes comprehensive deep geothermal use without spatial limitations. Geothermal development for heating in any geothermal play should consider the spatial distribution of heat demand clusters (“heat sinks”). Also, heating grid optimization or further coupling with other technologies such as heat pumps, seasonal heat storage or other renewables are not considered. In addition, aquifer heterogeneity and anisotropy might vary on scales that differ from the hexagon size applied in this study. Consequently, an even distribution of heat plants with a spacing of 4 km is not realistic and only chosen to reduce the complexity of our study, which should be addressed through comprehensive geophysical exploration and geological modeling when developing a geothermal play. Also, a thermal-hydraulic interference between projects is not considered, but can probably occur with the chosen distance and potentially leads to a decrease in the overall thermal power.

#### Volumetric production rates

The used volumetric production rate distributions only reflect the present-day knowledge and are based on the available dataset of the implemented wells. With each new well drilled and tested, the distributions will most likely need to be adjusted because of the reservoir heterogeneity. Also, the chosen distributions have their own uncertainties and were harmonized over large zones. This is a rough estimation, considering the heterogeneity of a carbonate reservoir, where an interplay of matrix, karstified, and fractured structures control the hydraulics^31,37^. Additionally, the equal flow rate distributions assume similar well design for each project within each zone. Even though the evolution of geothermal well completion over the last decades has been considered in drafting the distributions, the adjustment is of qualitative nature. Also, the impact of future advancements in drilling technology (extended reach, multi-laterals) on drilling cost and volumetric flow rates has not been considered in this study.

#### Temperatures

We used a production temperature that results from a comparative analysis of the static temperatures at the top reservoir from the geothermal information system GeotIS^33,34,44^ and the outflow temperatures from 33 wells in operation in the study area. The range of a linear regression can thus indicate the uncertainty of the calculated production temperatures, which is ±12 K. We have conducted this study within a theoretical framework that does not aim to predict specific thermal outputs for specific regions. For such more detailed predictions, it is recommended to also vary the temperatures within the corresponding uncertainties. Likewise, the assumed flowback temperature *T*_*back*_ represents a mean value from all active hydrogeothermal projects in the North Alpine Foreland in SE Germany^29^ and, depending on the heating grid type and future plans to employ centralized or decentralized heat pumps to further elevate thermal output, might differ from project to project.

#### Aquifer depths

We took the aquifer depth from the GeotIS model, which provides an accuracy of 100 m^33,44^. In regions without seismic (2D or 3D) coverage, however, higher deviations may also occur due to undetected displacements of faults. This also applies to the more southern regions in the study area, where the depths of subsidence are greater, which means that an increase in uncertainty is to be expected.

## Data Availability

Aquifer depth, temperature and basic production data are freely available^29,33,44^. All other relevant data are available from the authors upon request.
